# Population-genomic insights into emergence, crop adaptation and dissemination of *Pseudomonas syringae* pathogens

**DOI:** 10.1099/mgen.0.000089

**Published:** 2016-10-21

**Authors:** Caroline L. Monteil, Koji Yahara, David J. Studholme, Leonardos Mageiros, Guillaume Méric, Bryan Swingle, Cindy E. Morris, Boris A. Vinatzer, Samuel K. Sheppard

**Affiliations:** ^1^​ Institute of Life Science, College of Medicine, Swansea University, Swansea, UK; ^2^​ Department of Plant Pathology, Physiology, and Weed Science, Virginia Tech, Blacksburg, VA, USA; ^3^​ INRA, UR0407 Pathologie Végétale, Montfavet cedex, France; ^4^​ Laboratoire de Bioénergétique Cellulaire, Institut de Biosciences et Biotechnologies d'Aix-Marseille, CEA, 13108, Saint-Paul-lès-Durance, France; ^5^​ National Institute of Infectious Diseases, Tokyo, Japan; ^6^​ Biosciences, University of Exeter, Exeter, Devon, UK; ^7^​ The Milner Centre for Evolution, Department of Biology and Biotechnology, University of Bath, Claverton Down, Bath, UK; ^8^​ School of Integrative Plant Science, Section of Plant Pathology and Plant-Microbe Biology, Cornell University, Ithaca, NY, USA; ^9^​ Department of Zoology, University of Oxford, Oxford, UK

**Keywords:** Disease emergence, pathoadaptation, crop diseases, *Pseudomonas syringae*, type III secreted effectors

## Abstract

Many bacterial pathogens are well characterized but, in some cases, little is known about the populations from which they emerged. This limits understanding of the molecular mechanisms underlying disease. The crop pathogen *Pseudomonas syringae sensu lato* has been widely isolated from the environment, including wild plants and components of the water cycle, and causes disease in several economically important crops. Here, we compared genome sequences of 45 *P. syringae* crop pathogen outbreak strains with 69 closely related environmental isolates. Phylogenetic reconstruction revealed that crop pathogens emerged many times independently from environmental populations. Unexpectedly, differences in gene content between environmental populations and outbreak strains were minimal with most virulence genes present in both. However, a genome-wide association study identified a small number of genes, including the type III effector genes *hopQ1* and *hopD1*, to be associated with crop pathogens, but not with environmental populations, suggesting that this small group of genes may play an important role in crop disease emergence. Intriguingly, genome-wide analysis of homologous recombination revealed that the locus Psyr 0346, predicted to encode a protein that confers antibiotic resistance, has been frequently exchanged among lineages and thus may contribute to pathogen fitness. Finally, we found that isolates from diseased crops and from components of the water cycle, collected during the same crop disease epidemic, form a single population. This provides the strongest evidence yet that precipitation and irrigation water are an overlooked inoculum source for disease epidemics caused by *P. syringae*.

## Data Summary

We confirm all supporting data, code and protocols have been provided within the article or through supplementary data files. Sequencing reads have been submitted to the NCBI Small Read Archive as Bioproject PRJNA320409 with biosample accession numbers SAMN04942971 to SAMN04943055 and SAMN05301579 to SAMN05301583 and can be accessed at the following link: http://www.ncbi.nlm.nih.gov/bioproject?LinkName=sra_bioproject&from_uid=2500232.

## Impact Statement

Just like human diseases, new crop diseases emerge without warning and sometimes spread rapidly around the globe causing devastation. Where these pathogens originally came from is often unknown. The bacterial species *Pseudomonas syringae* consists of a group of genetically diverse bacteria including strains that are important crop pathogens as well as strains isolated from wild plants and components of the water cycle, such as clouds, rain and freshwater. The existence of these environmental strains, which are closely related to crop pathogens, suggests that crop pathogenic *P. syringae* possibly emerged from a diverse pre-existing *P. syringae* population that was present in the environment before the development of modern agriculture. Here, we found evidence for this hypothesis by sequencing and comparing the genomes of crop pathogenic and environmental strains, we inferred their evolutionary relationships and we identified genes with putative key roles in emergence of crop disease.

## Introduction

Successful disease prevention and management rely on a detailed understanding of the ecological and evolutionary processes driving disease emergence. In the case of bacterial crop diseases, a lot has been learned about crop pathogen virulence genes and their function (Lindeberg *et al.*, 2008; Tampakaki *et al.*, 2011; O'Brien *et al.*, 2011) but little is known about the genetic basis of crop disease emergence (Vinatzer *et al.*, 2014) and the conditions that promote it (Stukenbrock & McDonald, 2008). For diseases caused by host-restricted obligate pathogens such as *Puccinia striiformis* f. sp. *tritici* and *Puccinia graminis*. f. sp. *tritici*, the causal agents of stripe rust and stem rust, respectively (Chen, 2005; Singh *et al.*, 2011), these issues can be addressed relatively easily because dissemination patterns and ecology are restricted to one or a small number of plant hosts. Where infection can be caused by isolates in multiple environmental sources, it can be more difficult to pinpoint the source. For many human pathogens, the role of environmental reservoirs in disease epidemiology has been well described (Struve & Kogfelt, 2004; Whiley *et al.*, 2013; Grosso-Becerra *et al.*, 2014; Hazen *et al.*, 2015), but for facultative saprophytic crop pathogens, with environmental reservoirs, dissemination routes and interactions within multiple habitats are mostly uncharacterized (Woolhouse *et al.*, 2001; Johnson *et al.*, 2015).

In the past 10 years, MLSA studies have revealed considerable genetic diversity among environmental isolates that are closely related to epidemic, clonal crop-pathogenic lineages of the plant pathogen *Pseudomonas syringae* (*sensu lato*) (Morris *et al.*, 2008, 2010; Monteil *et al.*, 2012, 2014b). *P. syringae* is one of the economically most important bacterial crop pathogens and a well-characterized model species for molecular plant–microbe interactions (Hirano & Upper, 2000; O'Brien *et al.*, 2011). Environmental isolates have been collected from wild plants as well as non-plant reservoirs including soil, plant debris, and components of the water cycle including clouds, precipitation and surface water (Morris *et al.*, 2013; Berge *et al.*, 2014). Host range analysis revealed that some crop-pathogenic epidemic clones (referred to as ‘crop pathogens’ from here on) within *P. syringae*, such as the most common lineage of the tomato pathogen *P. syringae* pv. *tomato* (*Pto*), have a narrow host range limited to tomato (Cai *et al.*, 2011a, b). Conversely, lineages such as the cantaloupe pathogen *P. syringae* pv. *aptata* (*Pap*) have a broad host range, infecting various plant families (Morris *et al.*, 2000; Berge *et al.*, 2014). In the case of *Pto*, MLSA revealed the existence of closely related isolates from natural freshwater sources and recombination events between these environmental isolates, *Pto* and other crop pathogens (Monteil *et al.*, 2013). The environmental lineages were found to be equipped with some of the same virulence genes as the crop pathogen *Pto*, in particular genes coding for type III secretion (T3S) effectors, the best studied and most important class of virulence genes in *P. syringae* (Lindeberg *et al.*, 2008). Moreover, the environmental isolates had a wider host range than *Pto* but were less virulent on tomato (Monteil *et al.*, 2013). Taken together, these results are consistent with the evolution of highly virulent crop pathogens with a relatively narrow host range from a population of ancestors with a wider host range. This potentially occurs through the acquisition of genomic elements that promote virulence on the crop hosts but reduce virulence (or fitness) on other hosts. However, what these genomic elements might be, and whether they were acquired by horizontal gene transfer, remains unknown.

The increasing availability of large genomic datasets provides new opportunities for investigating pathotypes in multiple niches (Vinatzer *et al.*, 2014). By comparing the genomes of *P. syringae* crop pathogens and isolates from environmental reservoirs, it should be possible to identify the genetic basis of disease emergence and the genomic regions that are horizontally transferred between strains, in particular between crop pathogens and their environmental relatives. Therefore, we sequenced the genomes of 107 isolates of crop-pathogenic and environmental *P. syringae* and analysed them together with 86 publically available *P. syringae* genomes. We investigated two *P. syringae* phylogroups (Berge *et al.*, 2014) with contrasting host ranges (Buell *et al.*, 2003) and disease etiology (Feil *et al.*, 2005): phylogroup 1a, which includes *Pto* and other related crop pathogens and environmental isolates; and a subset of phylogroup 2d, for which we sampled exclusively closely related *Pap* isolates from diseased cantaloupe and the environment. Importantly, each phylogroup also includes one intensively studied model pathogen isolate: *P. syringae* pv. *tomato* (*Pto*) DC3000 in phylogroup 1a and *P. syringae* pv. *syringae* (*Psy*) B728a in phylogroup 2d. For both of these isolates, virulence traits have been investigated for decades and closed genome sequences are available (O'Brien *et al.*, 2011). Phylogenetic reconstruction, core and accessory genome analysis, and genome-wide association approaches were then used to characterize the evolutionary relationships between crop pathogens and environmental relatives, the population structure of these phylogroups and the genetic basis of crop adaptation (Sheppard *et al.*, 2013; Pascoe *et al.*, 2015). The results provide new insight into crop pathogen emergence, crop adaptation and pathogen dissemination.

## Methods

### Isolates and sequencing.

Genomes of 92 *P. syringae* isolates from phylogroups 1a and 2d (Berge *et al.*, 2014) were chosen for sequencing, whereby phylogroup 1a was sampled maximizing genetic diversity avoiding multiple crop pathogen strains with identical MLSA sequences, while for phylogroup 2d only one subset was sampled with isolates that were identical at two MLSA loci. These datasets were augmented with 12 genome sequences from the same phylogroups available in public databases, including reference genomes from *Pto* strain DC3000 (Buell *et al.*, 2003) and *Psy* strain B728a (Feil *et al.*, 2005). Most of the isolates sequenced in this study were described previously (Morris *et al.*, 2008; Morris *et al.*, 2010; Monteil *et al.*, 2012; Monteil *et al.*, 2014b) with 36 isolates collected from diseased crops (defined as cultivated lands) and 56 isolates collected from streams and rivers (11 isolates), precipitation (15 isolates), irrigation water (11 isolates) or epilithic biofilms (12 isolates) and leaf litter (seven isolates). Table S1 (available in the online Supplementary Material) provides a detailed list. Genomes representing other *P. syringae* phylogroups were included in the analyses: three of them were sequenced in this study while 86 genomes were downloaded from public databases to give a total of 193 genome sequences (Table S1).

For genomes sequenced in this study, DNA was extracted using the Gentra Puregene bacteria kit (Qiagen; cat. no.: 158567), according to the manufacturer’s instructions. The library preparation was performed with the Nextera XT DNA sample prep kit from Illumina following the manufacturer’s instructions for denaturing and normalization steps. High-throughput genome sequencing was performed using the 151×151 PE Rapid Run mode of a HiSeq 2500 sequencer (Illumina) using Illumina Truseq sequencing reagents. The quality of resulting sequencing reads was examined using FastQC (Andrews, 2010). TrimGalore (Krueger, 2015) was used to trim sequence reads and remove poor-quality data, using command-line options ‘-q 30 and -paired’. Illumina adapter sequences were removed using CutAdapt (Martin, 2011). Cleaned sequencing reads were assembled using the *de novo* assembly algorithm Velvet (Zerbino & Birney, 2008) (version 1.2.08). The value of *k* was optimized by assembling over a range of values and choosing the assembly with maximal N_50_. The minimum output contig size was set to 200 bp with the scaffolding option switched off, and all other program settings were left unchanged. The average number of contigs and the standard error in 95 newly sequenced *P. syringae* genomes was 474±32 for an average total assembled sequence size of 5 969 322±18 142 bp. Sequence reads in FastQ format deposited in the Sequence Read Archive (SRA) are available via BioProject accession PRJNA320409.

### Core and accessory genome.

Analysing core and accessory genome variation, genealogies and recombination patterns, we investigated the evolutionary relationships linking outbreak strains to their relatives in the environment. A reference pan-genome approach (Méric *et al.*, 2014) and gene-by-gene alignment (Sheppard *et al.*, 2012) was implemented using BIGSdb open source software (Jolley & Maiden, 2010). First, a reference gene list was assembled from four publicly available genomes, *Pto* DC3000 (Buell *et al.*, 2003) and *Psy* B728a (Feil *et al.*, 2005), *P. syringae* pv. *phaseolicola* 1448a (Joardar *et al.*, 2005) and the environmental strain CC1557 (Hockett *et al.*, 2014) (Table S1). The total number of genes in these isolates was 20 955 and after removal of 13 471 allelic variants that shared >70 % nucleotide similarity across ≥50 % of the genes' length, the final reference pan-genome list contained 7484 unique loci. Each locus was searched in the 193 genomes of all isolates using the blast algorithm and setting parameters for locus match to a minimum of 70 % sequence similarity over a minimum of 50 % of the query sequence length. The average core genome nucleotide sequence similarity within *P. syringae* is considerably higher than the blast match criteria. Therefore, these blast parameters ensure relatively low stringency for identifying homologous genes as in existing whole-genome MLST methodology (Jolley & Maiden, 2010; Sheppard *et al.*, 2012; Maiden *et al.*, 2013; Méric *et al.*, 2014). Consistent with whole-genome MLST (Berge *et al.*, 2014), a matrix was produced summarizing the presence/absence and allelic diversity of reference pan-genome genes, based upon these blast parameters. Each gene of the reference pan-genome that was not, or only partially, detected in a genome was indicated as missing or truncated, and this number was calculated at each locus for all *P. syringae* genomes. However, truncated gene sequences detected at the end or beginning of a contig were considered as present but were not counted as alleles. For each pair of isolates, the number of shared genes and alleles (identical sequences at a given locus over the whole sequence length) was calculated and the core genome for each species, and for the genus, was defined as the complement of genes that were present in all isolates.

### Population genetic structure.

Phylogenetic trees were reconstructed from the alignment of the core genome. The core genome was determined based on the 5619 coding sequences of the reference genome *Pto* DC3000 and its two plasmids. Genes in the core genome were aligned individually using mafft (Katoh & Toh, 2008) and concatenated to produce contiguous sequence alignments.

For analysis of the entire *P. syringae* species complex (193 genomes), core loci for which some of the sequences were truncated were kept in the analysis, which accounts for a total of 1889 genes. A recompiled version of FastTree 2.1.7 (Price *et al.*, 2010) was used to reconstruct an approximation of a maximum-likelihood tree. With this configuration, the minimum branch length was one substitution for every 2 000 000 bp (1000 times higher than the default FastTree parameters). The software was run with the Jukes–Cantor model of nucleotide evolution and gaps from truncated sequence alignments were considered as missing data. The tree was visualized and annotated using FigTree v1.4.2 (http://tree.bio.ed.ac.uk/software/figtree).

For the analysis of phylogroups 1a and 2d, trees were reconstructed from alignments including only non-truncated sequences for all core loci (respectively 810 and 2147 loci). Genealogies were inferred using ClonalFrame, a model-based approach for inference of microevolution in bacteria that accounts for recombination events that can disrupt phylogenetic reconstruction (Didelot & Falush, 2007). This program differentiates mutation and recombination events on each branch of the tree based on the density of polymorphisms. Clusters of polymorphisms are likely to have arisen from recombination, and scattered polymorphisms are likely to have arisen from mutation. The program was run with 20 000 burn-in iterations, followed by 50 000 and 100 000 sampling iterations for phylogroups 2d and 1a, respectively, until convergence. The consensus tree represents combined data from three independent runs with 75 % consensus required for inference of relatedness. Recombination events were defined as sequences with a length of >50 bp with a probability of recombination of 75 % over the length, reaching 95 % in at least one site.

Associations of lineages with isolation sources were investigated applying the HierBAPS clustering model (Corander *et al.*, 2004; Cheng *et al.*, 2013). This method reveals nested genetic population structures and any association of strain metadata with genetically divergent clusters and the substructure within them. Therefore, we were able to specify the genetic boundaries between *P. syringae* lineages at different resolutions and test if they were associated with a unique isolation source or not. The same alignments used for each tree reconstruction (whole *P. syringae* diversity, 1a or 2d) were used to infer genetically divergent clusters increasing levels of resolution from 1 to 4. The mixture partition was inferred setting the prior to 10 *k* panmictic subpopulations in which individuals were uniformly distributed and the structure, which maximizes the posterior distributions, was obtained using a stochastic search algorithm.

### Genome-wide association mapping.

We sought to determine the genetic basis of crop pathogen emergence by comparing genomes of crop pathogens and their relatives from other sources. We used a recently developed genome-wide association study (GWAS) method that is adapted for bacterial populations (Sheppard *et al.*, 2013; Pascoe *et al.*, 2015; Yahara *et al.*, 2016b) and excludes associations due to confounding population structure. The whole-genome sequence of each isolate was fragmented into unique overlapping 30 bp 'words'. For each word, the method examines the extent of association with the phenotype. To test the signiﬁcance of association of each word after controlling for the effect of population structure and clonal inheritance of genetic variants, here determined using ClonalFrame (Didelot & Falush, 2007), the method computes *P* values by comparing the observed association score with a null distribution of the score calculated through Monte Carlo simulations (Martins & Garland, 1991; Garland *et al.*, 2005). To account for multiple testing, only words with a probability below 5×10^−4^ were considered significant. The distribution of source-associated words for which homologues were found in reference genome *Pto* DC3000 (Buell *et al.*, 2003) was visualized using Artemis (Rutherford *et al.*, 2000) and DNAPlotter (Carver *et al.*, 2009) (see Fig. 3).

### Inference of homologous recombination.

The extent of homologous recombination was inferred between crop pathogens and related environmental isolates and ancestors of phylogroups 1a and 2d, to investigate genetic fluxes at a fine scale and the evolutionary origin of genes associated with disease outbreak populations. First, chromosome painting (Lawson *et al.*, 2012; Yahara *et al.*, 2013) was used to build a co-ancestry matrix summarizing the number of recombination-derived ‘chunks’ of DNA from each donor to each recipient isolate. Using this matrix, fineSTRUCTURE (Lawson *et al.*, 2012) was used to conduct model-based clustering of individuals by a Bayesian Markov chain Monte Carlo (MCMC) approach that explores the space of possible partitions. In parallel, we applied the Ordered Painting approach (Yahara *et al.*, 2014) to identify hotspots of recombination within phylogroups. In this method, the extent of genealogical changes for a specific site due to recombination compared with the average genome genealogy is represented by the distance statistic *H_i_* representing recombination hotness at each polymorphic site *i* (Yahara *et al.*, 2016a). These atypical changes, for example, above the top percentile of the whole-genome distribution, are expected to indicate hotspots of recombination.

## Results

### Population structure of the *P. syringae* species complex

Before determining the population structure of *P. syringae sensu lato*, we determined whether the 193 *P. syringae* genome sequences (sequenced in this study or publically available) formed a clade that is distinct from genomes of other *Pseudomonas* species. A genealogy of 629 available *Pseudomonas* species genomes was reconstructed based on 52 ribosomal protein gene sequences (Tables S1 and S2). The phylogenetic tree confirmed *P. syringae* as a largely monophyletic species complex. The one exception was phylogroup 13 [isolate UB246 (Berge *et al.*, 2014)], which clustered with *Pseudomomas fluorescens* based on population structure analysis inferred with BAPS, a Bayesian statistical clustering method (Fig. S1).

**Fig. 1.**
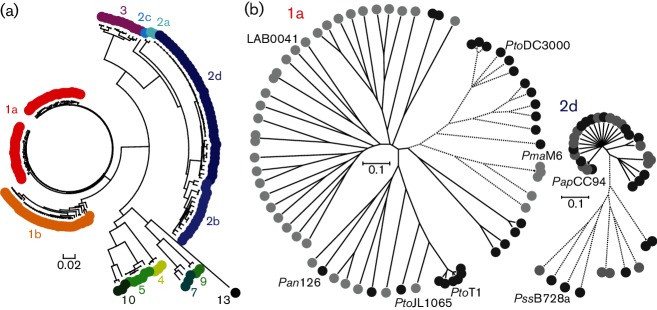
Population structure of 193 isolates from the *P. syringae* species complex. (a) Unrooted phylogenetic tree reconstructed from 1889 genes detected in all isolates using an approximation of the maximum-likelihood algorithm and the GTR model for nucleotide substitution. Bar, the number of substitutions per site. Isolates are coloured according to the monophyletic groups described by Berge *et al.* (2014). (b) Genealogies inferred by ClonalFrame (Didelot & Falush, 2007) for phylogroups 1a and 2d based only on those genes with no evidence of homologous recombination (810 and 2293 genes, respectively). The trees are drawn to scale, with branch lengths corresponding to the number of substitutions per site. Dark and light grey circles symbolize isolation sources: crop pathogens and environmental strains, respectively. Solid and dotted branches symbolize subpopulations within each phylogroup determined with the first level of HierBAPS hierarchy (Corander *et al.*, 2008; Cheng *et al.*, 2013).

To analyse evolutionary relationships with greater resolution within the *P. syringae* species complex, a core genome of the 193 isolates (Table S2) was determined by aligning all genome sequences with the annotated genes of the fully sequenced and annotated reference genome *Pto* DC3000 (Buell *et al.*, 2003). The *P. syringae* genealogy reconstructed from 1889 core gene loci (a total of 108 393 bp for which 38 484 were variable sites) confirmed genealogies previously inferred by MLSA and by previous core genome analysis (Berge *et al.*, 2014). Importantly for the goal of this study, it also revealed recent common ancestry of all lineages within phylogroups 1a and 2d, respectively (Fig. 1a). To determine the extent to which these phylogroups reflected distinct genetically divergent clusters within the *P. syringae* species complex, we performed a hierarchical clustering analysis of the core genome using the HierBAPS method that estimates nested population structures (Cheng *et al.*, 2013). Based on clustering at the lowest level of resolution, clade designations were congruent with most phylogroups, including phylogroups 2d and 1a.

To investigate the role of environmental reservoirs in the emergence of crop pathogens, we then focused on phylogroup 1a, for which we had sequenced representative isolates of all crop pathogens available to us and all available environmental relatives in order to include as much genetic diversity as possible. Individual genealogies were built and Bayesian analysis of population structures (Cheng *et al.*, 2013) was performed. Since homologous recombination may have had an impact on population genetic structure, clonal relationships of crop pathogens and their relatives were first investigated using ClonalFrame (Didelot & Falush, 2007), which accounts for recombination when reconstructing the genealogy. At the lowest degree of resolution, the population structure analysis of all core genes identified three genetic clusters, each containing both crop pathogens and environmental relatives (Fig. 1b). The ClonalFrame analysis also revealed that some monophyletic groups of lineages within these clusters correspond to one isolation source only. For example, the *Pto* DC3000 crop pathogen clusters only with other crop pathogens, and the LAB0041 isolate from an alpine epthilic biofilm clusters only with other environmental isolates. Importantly, however, several crop pathogens, such as the tomato pathogens *Pto* T1 and *Pto* JL1065 and the snapdragon pathogen *Pan* 126, are interspersed with lineages from rain or surface water within the same group. This shared ancestry between some crop pathogens and some environmental isolates, together with the long external, short internal branches of the ClonalFrame tree (Fig. 1b), and a high degree of reticulation in the NeighborNet network (Fig. S2a), are all consistent with a scenario of multiple emergences of different crop pathogens and environmental lineages from recombining ancestral populations.

For phylogroup 2d, we investigated the different components of the water cycle in pathogen dissemination. Isolates from diseased crops, collected during a cantaloupe blight epidemic in France, were sequenced together with isolates from precipitation, surface water, irrigation water and ground water (Morris *et al.*, 2000; Morris *et al.*, 2008). Specifically, we chose isolates that were identical at two MLSA loci and we wanted to determine whether crop and environmental isolates would cluster together, or separately, based on core genome sequences. ClonalFrame and population structure analysis revealed that most of these isolates clustered together with a star-like genealogy without any separation between crop isolates and environmental isolates. The genomes of crop and environmental isolates in the first cluster were extremely similar to each other (average nucleotide identities ranging from 99.50 to 99.97 % within each clade). To confirm these results, sequencing reads were aligned against the *Psy* B728a genome and SNPs were identified. This approach revealed that some of the crop isolates differed from their most similar environmental relatives by as few as three SNPs per million base pairs (data not shown), suggesting very recent exchanges of *P. syringae* between cantaloupes and water cycle components. Moreover, ClonalFrame showed that some core genes experienced recent homologous recombination between crop and environmental isolates, which was supported by the NeighborNet network shown in Fig. S2b.

### Core and accessory genome variation and pathogen emergence in phylogroups 2d and 1a

**Fig. 2.**
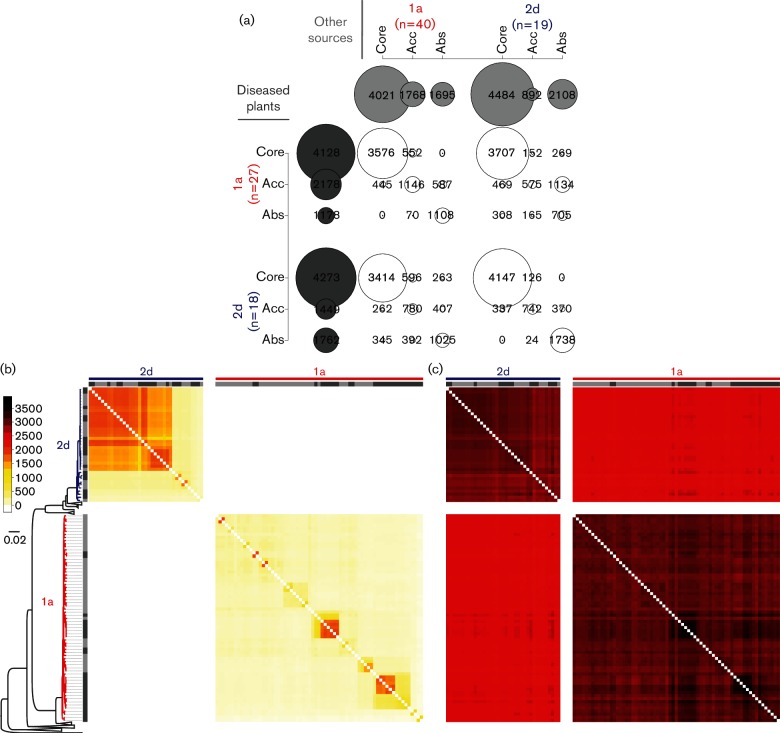
Core genome similarity and accessory genome variation within and between phylogroups 2d and 1a. (a) Overlap between the core and accessory genomes calculated for phylogroups 1a and 2d. Core genes (Core), accessory genes (Acc) and absent genes (Abs) were defined as genes detected in 100 % of the isolates, detected in less than 100 % but more than 0 % of the isolates, and absent in 100 % of the isolates, respectively. The radius of each circle is proportional to the number of detected genes. Dark grey circles represent genes from strains isolated from diseased crops while light grey circles represent genes from environmental isolates. White circles represent overlapping genes from both populations. Matrices show pairwise comparison of core genome similarity (b) and accessory genome variation (c) between 104 isolates ordered according to the phylogenetic tree presented in Fig. 1. Entire matrices with all 193 *P. syringae* isolates are shown in Fig. S4. Dark grey boxes symbolize crop pathogens while isolates from other sources are symbolized by light grey boxes. Heatmap colours ranging from white, through yellow, red to black represent values from the lowest to the highest number of shared alleles or genes, in the first and second heatmap, respectively. The minimum number of shared alleles in the core genome ranged from 0 to 3010 while the minimum number of shared accessory genes ranged from 1105 to 2508.

Genome-wide genetic differences between crop pathogens and environmental isolates were investigated using a reference pan-genome approach (Méric *et al.*, 2014). In short, four fully assembled and annotated genome sequences from four different phylogroups were chosen as references: *Pto* DC3000 (Buell *et al.*, 2003), *Psy* B728a (Feil *et al.*, 2005), *Pph* 1448a (Joardar *et al.*, 2005) and CC1557 (Hockett *et al.*, 2014). The pan-genome of these four reference genomes, defined as the total set of gene families present in the four genomes, was then determined and found to consist of 7484 unique genes. Finally, the reference pan-genome was aligned against all the other genomes. This analysis revealed that every genome in phylogroup 1a contained a set of 3576 genes of the reference pan-genome, thus representing the 1a core genome. Every genome of phylogroup 2d contained a set of 4147 core genes representing the 2d core genome. In total, 3062 genes were present in both phylogroups, representing their combined core genome. At the remaining 4422 loci, genes were either present or absent, presenting the combined accessory genome (Fig. 2a). Phylogroup 2d had a lower average p-distance between allele pairs, and on average fewer unique alleles per gene: 0.201±0.001 compared to 0.480±0.003 for phylogroup 1a (Student’s *t*-test, *P*<0.001, Fig. S3a,b). Note that differences in the genetic diversity of phylogroups 2d and 1a reflect our sampling strategy, maximizing genetic diversity for phylogroup 1a while prioritizing a single genetic lineage for phylogroup 2d.

To determine the extent of genes associated with isolation sources, we compared gene content of crop pathogens and environmental isolates. It is striking that none of the genes that are core to crop pathogen populations are absent in environmental populations, and that none of the genes that are core to environmental populations are absent in crop pathogen populations (Fig. 2a). This suggests that there is weak ecological differentiation between crop pathogens and their environmental relatives. Moreover, for both phylogroups the majority of accessory genes present in environmental isolates are also present in crop pathogens and vice versa. This result reveals that there is no strong barrier to gene flow between environmental isolates and crop pathogens. Importantly, about 61 % of T3S effector genes detected in crop pathogen populations were present in environmental relatives as well (Table S3, Fig. S5).

Pairwise genome comparisons showed that patterns of core genome allelic similarity (Fig. 2b) and accessory genome similarity, measured as similarity with regard to presence and absence of accessory genes (Fig. 2c), reflected genealogies rather than isolation host or source (crop versus environment). Extending this analysis beyond phylogroups 1a and 2d to *P. syringae* genome sequences in phylogroups 2b and 3 confirmed the same trend (Fig. S4). Therefore, pairwise genome comparisons confirmed the absence of strong barriers to gene flow between crop pathogens and environmental relatives as well as between pathogens of different hosts.

### Candidate genes associated with pathoadaption

**Fig. 3.**
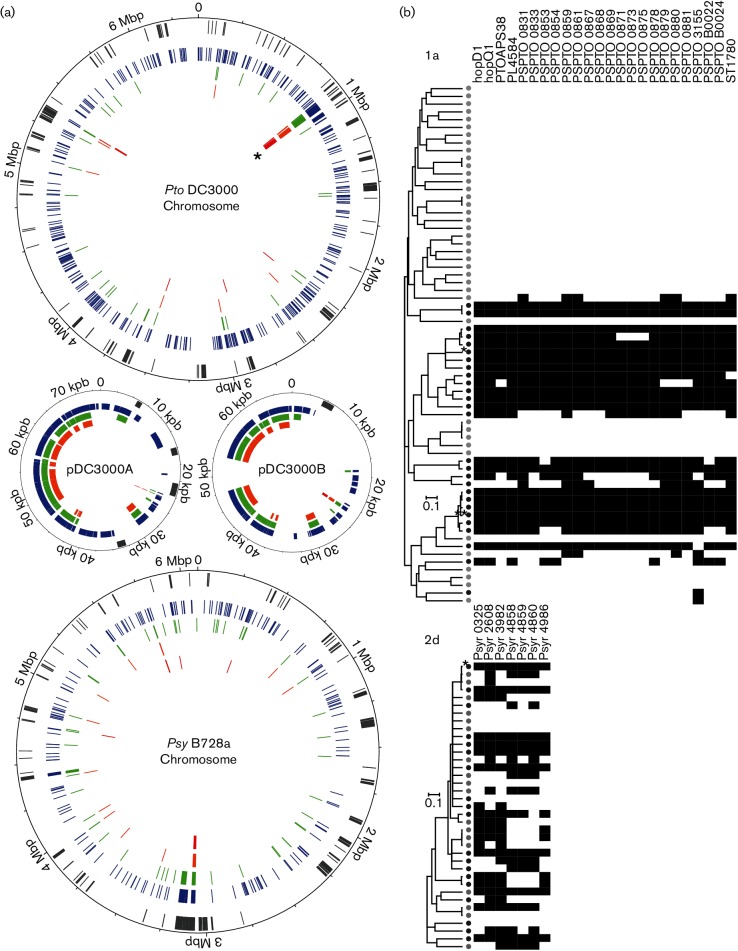
Distribution of genes associated with *P. syringae* crop pathogen strains within reference genomes and within populations. (a) The 30 bp long words that were found to be significantly associated with crop pathogens in either phylogroup 1a or 2d were mapped on the genome of the reference crop pathogens *Pto* DC3000 and *Psy* B728a, respectively. A total of 73 299 and 5970 crop pathogen-associated words in *Pto* DC3000 and *Psy* B728a were distributed in 571 and 222 genes, respectively. The list of these genes and the distribution of mapped words is given in Table S4. For each chromosome and plasmid, the first grey circle represents virulence genes (listed in Lindeberg *et al.*, 2008). The next four coloured circles correspond to the genes in which words were mapped from the highest to the lowest probability (blue, green, orange and red corresponding to *P* value cutoffs of 5×10^−^^4^, 5×10^−^^5^, 5×10^−^^6^ and 5×10^−^^7^, respectively). The 25 kb region containing *hopQ1* and *hopD1* described in the text is indicated with an asterisk. (b) Heatmaps showing the presence of genes for which at least one word was significantly associated with crop pathogens with a probability inferior or equal to 5×10^−^^7^. Black boxes denote that at least one word was mapped for the corresponding isolate and gene, while white boxes denote that not a single word was present with this probability. Isolates were organized following the core genome trees built with ClonalFrame (Didelot & Falush, 2007) in Fig. 1(b). The trees are drawn to scale, with branch lengths proportional to the number of substitutions per site. Dark and light grey circles symbolize crop pathogen strains and environmental isolates, respectively.

Although the previous analyses clearly showed that crop pathogens are not genetically isolated from their environmental relatives, it is still possible that at least a small set of genes, or alleles, may be more frequently associated with either crop pathogens or environmental isolates. This would suggest that crop pathogens are adapted to a pathogenic lifestyle (pathoadaptation) that is different from the adaptation of environmental isolates to a lifecycle in non-agricultural environments. Therefore, genetic elements overrepresented either in crop pathogen strains or in environmental isolates were determined using a GWAS (Sheppard *et al.*, 2013; Pascoe *et al.*, 2015), which identifies 30 bp DNA sequences (words) in the core and accessory genome, taking into account the clonal frame and vertical inheritance and core genes. Based on the analysis of 67 and 37 isolates within phylogroups 1a and 2d, 73 299 and 5970 words, respectively, were identified that were over-represented in crop pathogens. These mapped to 571 genes in phylogroup 1a and 222 genes in phylogroup 2d, 74 % of which are annotated with a putative function (Table S4). Genes containing pathogen-associated elements were mapped to reference genomes of the crop pathogens *Pto* DC3000 and *Psy* B728a, for 1a and 2d isolates, respectively, using Artemis (Rutherford *et al.*, 2000) and DNAPlotter (Carver *et al.*, 2009) (Fig. 3a). Associated genes were dispersed across the genome with evidence of seven (phylogroup 1a) and five (phylogroup 2d) hotspots of strong pathogen association, with a *P* value less than 5×10^−^^6^.

For phylogroup 1a isolates, only 7 % of the mapped words were associated with known virulence genes, with 22 % of the total associated words mapping to a single 25 kb region consisting of 21 adjacent genes of the *Pto* DC3000 genome (Fig. 3a). This included the T3S effector genes *hopD1* and *hopQ1* (Lindeberg *et al.*, 2008). These genes were present in all but two of crop pathogen isolates and absent from all environmental isolates (Fig. 3b). Importantly, after aligning raw reads of these two isolates against the *Pto* DC300 genome (data not shown), relic fragments of *hopD1* and *hopQ1* were even detected in the only two crop pathogens, *P. syringae* pv. *apii* BS252 and *P. syringae* pv. *antirrhini* 126, that did not contain the intact genes. Other crop pathogen-associated hotspots in phylogroup 1a contained genes encoding putative proteins related to: (i) replication, integration, recombination and repair of DNA; (ii) carbohydrate, lipid and amino acid transport; and (iii) energy production and conservation (Table S4).

Interestingly, no T3S effector genes were associated with crop pathogen isolates in phylogroup 2d and, compared to the 1a phylogroup, the repertoire of T3S effector genes was generally smaller in phylogroup 2d (Fig. S5). Predicted functions of genes that were significantly associated with crop pathogen isolates in 2d were: (i) DNA transcription and translation regulation; (ii) uptake of sparse substrates linked to TonB-dependent transporters; (iii) the conversion of energy into storage molecules; (iv) secondary metabolite production and export; and (v) type I secretion systems (Table S4). Note that sampling within phylogroup 2d focused on a subset of very similar isolates to address questions about pathogen dissemination. Additional sampling would be necessary for more robust inference of pathogen associations in this lineage.

### Homologous recombination and pathoadaptation

In recombining bacteria, the acquisition of DNA from other lineages can confer novel functions, such as those related to pathoadaptation (Ochman *et al.*, 2000). ClonalFrame analysis and patterns of reticulation using simple NeighborNet genealogical reconstructions (Fig. S2) suggested recent homologous recombination among various crop pathogens and environmental isolates. A more detailed analysis was thus carried out to investigate inter- and intra-phylogroup homologous recombination and to quantify recombination landscapes across the genome.

**Fig. 4.**
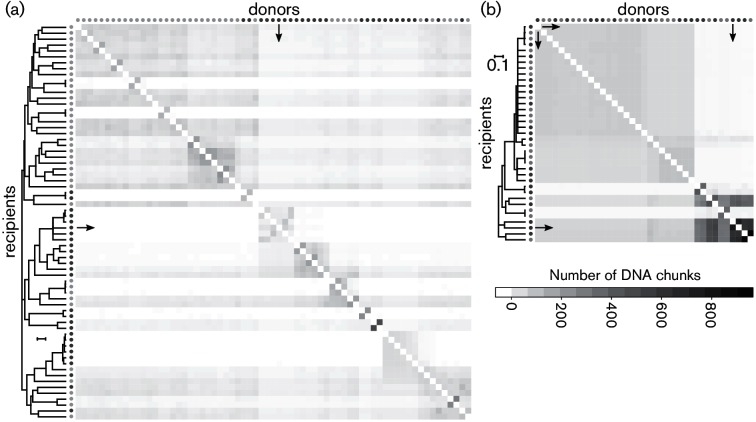
Inference of genetic fluxes within *P. syringae* populations. Co-ancestry matrices were determined by Chromosome Painting and fineSTRUCTURE for phylogroups 1a (a) and 2d (b). The expected number of ‘chunks’ imported from a donor genome (column) to a recipient genome (row) is given by the colour of each cell of the matrices. The trees are drawn to scale, with branch lengths proportional to the number of substitutions per site. Arrows in (a) point to *Pto* DC3000, while arrows in (b) point to *Pap* CC94 and *Psy* B728a at the top and bottom of the tree, respectively. Dark and light grey circles symbolize crop pathogen strains and environmental isolates, respectively.

Gene flow within and between phylogroups was quantified by characterizing DNA donated and received among the strains using chromosome painting (Yahara *et al.*, 2013). The number of recombination-derived chunks of DNA, defined as genetic material donated from a nearest ‘donor’ to a ‘recipient’ haplotype, was summarized into a co-ancestry matrix. A co-ancestry matrix with all 1a and 2d strains confirmed the barrier to gene flow between the two phylogroups for which the number of DNA chunks was under one per genome on average. However, gene flow was observed within each phylogroup (Fig. 4). Both co-ancestry matrices not only showed admixture between ancestors of all lineages but also showed that gene flow occurred between crop pathogens and environmental isolates in both directions, as for *Pap* CC94 (Fig. 4b). However, in phylogroup 1a isolates, gene flow was asymmetrical with some lineages being principally donors or recipients. For example, *Pto* DC3000 was mostly a donor and not a recipient to isolates from both isolation sources (Fig. 4a) in contrast to *Psy* B728a from phylogroup 2d, which received twice as many DNA chunks from ancestors of other crop and environmental isolates compared to what the strain donated to other strains (Fig. 4b).

**Fig. 5.**
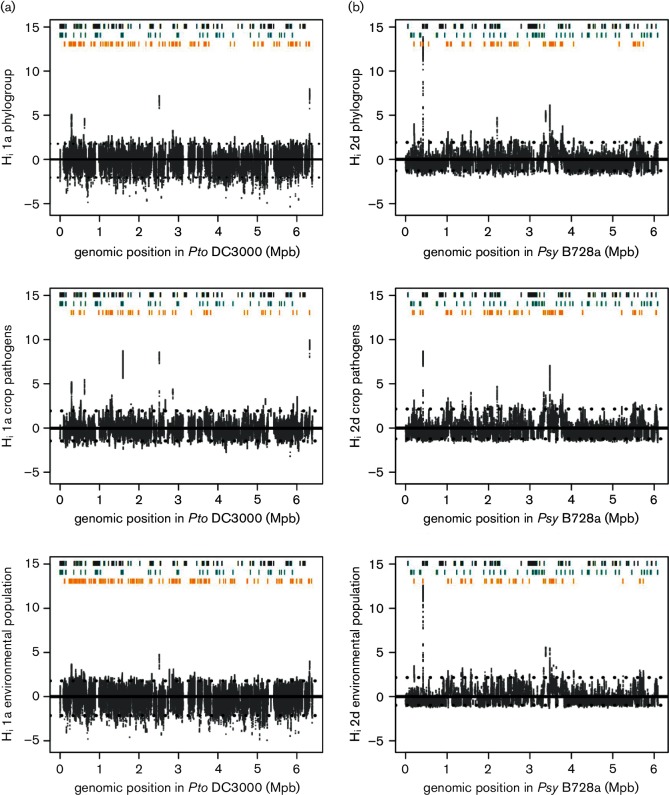
Inference of homologous recombination hotspots within *P. syringae* populations. Homologous recombination hotspots were inferred in phylogroups 1a (a) and 2d (b) as described by Yahara *et al.* (2014). A total of 177 790 and 141 997 SNPs were used, respectively, representing the chromosome of each reference genome. For each group, the extent of recombination was estimated from the whole phylogroup, from the crop pathogen strains only and, finally, considering only isolates from environmental reservoirs. The *x*-axis indicates the position in the reference genomes *Pto* DC3000 (Buell *et al.*, 2003) and *Psy* B728a (Feil *et al.*, 2005). The *y*-axis indicates the empirical distribution of the distance statistic *H_i_* representing the intensity of normalized recombination. The solid line represents the average value of *H_i_* in a genome The dotted lines represent the top and bottom 2.5 percentiles. Grey lines represent virulence genes (Lindeberg *et al.*, 2008). Turquoise lines represent genes associated with being a crop pathogen using the GWAS approach (Table S4). Orange lines represent genes associated with hotspots of recombination. The genes in the regions showing more intense recombination are given in Table S5.

In parallel to characterizing the direction of gene flow, recombination hotspots across the genome were identified based upon a per-site estimate of intensity of recombination (*H_i_*) (Yahara *et al.*, 2016a), which refers to a normalized value quantifying the extent of genealogical changes due to recombination compared to the average genealogy (Fig. 5). For phylogroups 2d and 1a, respectively, 144 and 244 recombining genes had *H_i_* values in the upper 2.5 % for at least one base position (Table S5). Evidence for a role of recombination in pathoadaptation was seen in phylogroup 1a where 72 genes recombined in crop pathogens. A total of 8 % were known virulence genes, including genes coding for T3S effectors (*hopAA1-1*, *hopAH1* and *hopB1*), T4 pili, chemotaxis, pioverdine production and levansucrase (*lsc-1*). The highest rates of recombination in crop pathogens were found for those genes that were also identified as hotspots of recombination in environmental isolates and were mostly associated with hypothetical proteins or a peptide ABC transporter permease (*PSPTO 265*, *561*, *2271*, *2587* and *5552*). In phylogroup 2d, 116 genes recombined in crop isolates and were mainly associated with metabolism, regulators (*lysR*, *lclR*, *tetR*), transporters and the type 1 secretion system (involving TolC and HlyD proteins), while the 23 genes recombining in environmental isolates were associated with other functions among which were several T3S effectors and structural components (e.g. *hopM1*, *hrcN*, *hrpQ*, *hrcV* and *hrpK1*). As observed within phylogroup 1a, hotspots of recombination in 2d isolates from crops were sometimes hotspots in environmental isolates as well. Hotspots in 2d were associated with genes coding for hypothetical proteins (PSYR 392 and 393), a tRNA-dihydroudine synthetase A (PSYR 1936) and a binding-protein dependent transport system (PSYR 2903 and 2904). Importantly, 18 orthologous genes were found to be recombining in both phylogroups, some of them coding for ABC transporters, proteins involved in antibiotic resistance (PSPTO 3132, 3302 and 5191), chemotaxis, extracellular solute binding (PSPTO 2962 and 3302), glutamate racemase (*murI*), porins (*oprD*) and a heavy metal translocating ATPase (*cad A_1*) (Table S5).

Finally, we sought to identify those genes associated with crop pathogens (based on GWAS) and hotspots of recombination, and determine whether they have been previously characterized as virulence genes. Comparisons flagged the PSPTO 5191/PSYR 0346 gene, a member of the AcrB/AcrD/AcrF family of membrane proteins, implicated in multiple antibiotic resistance in *Salmonella typhimurium* (Piddock, 2006), that is not only recombining in both phylogroups but also associated with pathoadaptation in 2d. Other genes associated with pathoadaptation in phylogroup 2d were also recombination hotspots in crop pathogens, such as PSYR 195, 336, 1992 and 3131 coding for a hypothetical protein, an outer membrane autotransporter barrel, a zinc-containing alcohol dehydrogenase superfamily protein and a secretion protein HlyD, respectively. However, only PSYR 1794 and PSYR 3151 coding for a non-ribosomal peptide and the protein E of a type II secretion system, respectively, were also known virulence genes (Lindeberg *et al.*, 2008).

In phylogroup 1a, the T3S effector gene *hopAH1* was the only known virulence gene located within a recombination hotspot and that had also been associated with pathoadaptation in GWAS. Other crop pathogen-associated genes that are not known virulence genes corresponded to hotspots of homologous recombination, including the ferric iron reductase protein *fhuF*, an acyltransferase *PSPTO 0997* (COG accession COG1835) and a transcriptional regulator from the GntR family (SMART accession smart00895).

## Discussion

The bacterial plant pathogen *P. syringae* is well known as a model organism to study the molecular basis of plant–microbe interactions (Alfano & Collmer, 2004; Xin & He, 2013). Moreover, there is no other bacterial plant pathogen species for which so much is known about genetic diversity outside of agricultural environments (Morris *et al.*, 2008, Morris *et al.*, 2010; Monteil *et al.*, 2012; Berge *et al.*, 2014), which has made *P. syringae* a model for studying crop pathogen emergence as well (Mohr *et al.*, 2008; Yan *et al.*, 2008; Cai *et al.*, 2011a, b; Diallo *et al.*, 2012; Morris *et al.*, 2013; Bartoli *et al.*, 2015). Here, we applied for the first time a population genomics approach to *P. syringae* crop pathogens and their close environmental relatives and gained new insight into crop pathogen ancestry, emergence, crop adaptation and dissemination.

Phylogenetic reconstruction based on a small number of genes had already suggested that epidemic *P. syringae* crop pathogens in phylogroup 1a, such as *Pto*, have close relatives in non-agricultural environments (Monteil *et al.*, 2013). After sequencing the genomes of representative crop pathogens and their environmental relatives in phylogroups 1a and 2d, we have now shown that several crop pathogens, including *Pto* T1 and *Pap* CC94, are more closely related to environmental isolates than to other crop pathogens. Moreover, by investigating patterns of recombination and population structure at the whole-genome level within each phylogroup, we show that several crop pathogens emerged from an ancestral recombining population independently from each other.

A fundamental question is when these emergence events occurred. Accurate molecular clock estimates are not possible within the sample frame of this study as it does not include a longitudinal sample. However, almost identical pathogen isolates have been sampled from crops dozens of years apart (Cai *et al.*, 2011a; Clarke *et al.*, 2015) and this is consistent with a mutation rate as low as one mutation per million base pairs per year. Therefore, considering that some of the *P. syringae* crop pathogen lineages in phylogroup 1a have diverged substantially from each other, more than one mutation per 1000 bp in some MLSA loci, it is likely that their most recent ancestor existed before humans started domesticating crop plants and before the advent of agriculture 5000–10 000 years ago. Therefore, the inferred ancestral population may have existed in non-agricultural plant communities and environmental reservoirs.

For phylogroup 2d, our analysis focused on a subset of isolates selected based on their identity at two MLSA loci excluding most of the genetic diversity that is known to exist in this phylogroup. This sample frame allowed comparison of contemporaneous isolates collected from diseased cantaloupe, sampled during a cantaloupe blight epidemic in France, and their closest relatives isolated from irrigation water, precipitation and ground water. Based on whole-genome analysis, isolates with almost identical genome sequences clustered together despite their different isolation sources and with some isolates from cantaloupes collected as far apart as 350 km. This result is consistent with frequent migration events between cantaloupe production fields and components of the water cycle and suggests that rain and irrigation water are involved in the dissemination of crop-pathogenic *P. syringae* between geographically distant fields. Although *P. syringae* had been reported in rain before (Constantinidou *et al.*, 1990; Morris *et al.*, 2008; Morris *et al.*, 2010; Monteil *et al.*, 2014a), no strong genetic linkage between the presence of *P. syringae* in rain or irrigation water on the one hand and *P. syringae* isolated from diseased crops on the other was possible without genome sequences. Additionally, in the specific case of phylogroup 2d isolates, which have a wide host range (Morris *et al.*, 2000), rain and irrigation water may also be the original inoculum source of epidemics by transporting the pathogen from colonized wild and crop plants over long distances to crop fields and starting new outbreaks. This has important implications for crop disease prevention programmes since it is commonplace to link new *P. syringae* disease outbreaks to contaminated seed and nearby weeds but not to components of the water cycle or irrigation water (McCarter *et al.*, 1983; Gitaitis & Walcott, 2007).

To understand the molecular basis of crop disease emergence, it is necessary to determine what differentiates epidemic crop pathogen isolates from their close relatives that are not epidemic pathogens. Bulk shotgun sequencing and virulence analysis of a small number of environmental isolates in phylogroup 1a had already revealed that these isolates contain well-known virulence genes such as T3S effector genes and that some of the environmental isolates are almost as virulent as *Pto* on tomato and other plant species (Monteil *et al.*, 2013). By extending comparison of gene content to multiple whole genomes, we show here that all environmental relatives in phylogroup 1a are equipped with T3S systems and with repertoires of T3S effectors and other virulence genes similar to those of crop pathogens. Moreover, extending our virulence assays to all environmental relatives in phylogroup 1a, we confirmed that these isolates are all pathogenic on tomato, although less virulent than *Pto* (data not shown). We thus conclude that although the analysed environmental isolates were originally mostly collected from water, they appear to be adapted to life in association with plants and they possibly are pathogens of wild plants. These observations raise many questions about the role of wild plants and crop plants in the emergence and diversification of virulence traits in plant pathogenic populations. In particular, considering the genetic diversity of virulence-gene-equipped environmental populations, it is not clear why there are not more frequent emergence events. For example, there is only a single *Pto* lineage that has spread successfully worldwide on tomato (Cai *et al.*, 2011a). It is therefore possible that there is a genetic barrier to emergence whereby only rare combinations of virulence genes allow emergence of an epidemic clone.

To test this hypothesis, a GWAS (Sheppard *et al.*, 2013) was performed to identify genomic regions that show a statistically significant association with crop pathogens. Intriguingly, only two of the 58 T3S effector genes, *hopD1* and *hopQ1*, were found to be pathogen-associated in phylogroup 1a and not a single T3S effector was found to be pathogen-associated in phylogroup 2d. Like some other T3S effectors, HopD1 interferes with the immune response triggered by other effectors (Block *et al.*, 2014) and HopQ1 interferes with immunity triggered by microbial-associated molecular patterns, specifically the immune response triggered by the bacterial flagellum (Li *et al.*, 2013a, b; Hann *et al.*, 2014). Moreover, just as for many other effectors, deletion of either *hopQ1* or *hopD1* from *Pto* DC3000 has been shown to reduce bacterial growth on some plant genotypes under laboratory conditions (Wei *et al.*, 2007). Therefore, the fact that only *hopQ1* and *hopD1* were identified as crop pathogen-associated in the GWAS suggests that the specific contribution to virulence by these two effectors is in some way more relevant in the life cycle of epidemic crop pathogens compared to the life cycle of bacteria associated with non-agricultural environments, while the other T3S effectors that are more evenly shared by crop pathogens and their environmental relatives play a role in fitness in agricultural as well as non-agricultural environments.

In phylogroup 2d, although our sampling strategy may have inflated associations, the GWAS revealed fewer statistically significant associations with source of isolation with lower *P* values compared to associations in phylogroup 1a. In particular, the distribution of the top GWAS hits within the group of most closely related isolates (Fig. 3b) showed no clear association at all. This is probably a result of our sampling frame and is consistent with our earlier conclusion that the sequenced 2d isolates represent a single population of *P. syringae* that regularly transfers between cantaloupe, other plant hosts and components of the water cycle.

Finally, our previous analysis of phylogroup 1a isolates (Monteil *et al.*, 2013), and analysis of the kiwifruit pathogen *P. syringae* pv. *actinidiae*, suggested that crop pathogens emerge from recombining *P. syringae* populations. While it has been suggested that this recombination mainly occurs within *P. syringae* pathogen populations specific to a host plant species, such as kiwifruit (McCann *et al.*, 2013), we provide evidence that the recombining *P. syringae* populations may not be host-specific and could include environmental *P. syringae* residing, for example, in wild plants and in decaying plant material, where *P. syringae* populations reach densities as high as 10^6^ c.f.u. g^–1^ (Monteil *et al.*, 2012). Importantly, using quantitative analysis of homologous recombination, we demonstrate that recombination between crop pathogens and their environmental relatives is as frequent as recombination between isolates within either niche (Fig. 4). This result reveals that *P. syringae* crop pathogens can acquire new genes from environmental populations and vice versa, possibly including potential virulence genes, which might exist at low frequency in environmental populations. In fact, although *hopD1* and *hopQ1* were not found in the environmental isolates analysed here, they may exist at low frequencies in these populations. One possible emergence scenario is that when a strain receives these genes in a recombination event from a donor, its fitness on crops increases, and it emerges as a highly virulent crop pathogen. Surprisingly, however, *hopAH1* was the only T3S effector located in one of the identified hotspots of homologous recombination while *hopD1* and *hopQ1* were not. Their exclusive presence in crop pathogens that are phylogenetically distinct and their absence from closely related environmental isolates is nonetheless consistent with acquisition by horizontal gene transfer. This conclusion is supported by inferred phylogenies based on *hopD1* and on *hopQ1* (Fig. S6) that were incongruent with the core genome tree, suggesting that these genes were in fact subject to recombination.

Among the genes found to be located within hotspots of recombination in both analysed phylogroups, and more frequently present in crop pathogen isolates than environmental isolates, was the gene with locus tag PSPTO 5191/PSYR 0346. Functional predictions using blast p of the translated sequences identified strong signatures of a conserved domain specific of the acriflavin resistance protein family (Pfam accession PF00873, E value 4.03×10^–143^; TIGRFAM00915, 8.04×10^–81^, >90 % length of sequence). Therefore, this putative membrane protein could be part of an aminoglycoside efflux system involved in either toxin production or resistance processes. This gene has been implicated in multiple antibiotic resistances in human pathogens such as *Salmonella* Typhimurium (Piddock, 2006) but its potential role in conferring resistance to antibiotics and/or other agropesticides in *P. syringae* is unknown. It remains the case that recombination was found to be most frequent between closely related isolates with little genetic exchange between 1a and 2d isolates, consistent with well-known homology dependence of recombination (Hanage *et al.*, 2009).

In conclusion, the extensive comparison of multiple *P. syringae* crop pathogens and their environmental relatives using several population genomics approaches clearly showed that not only does *P. syringae* frequently move between crop hosts and non-agricultural environments, but also *P. syringae* genes move just as frequently between one crop pathogen and the other as between crop pathogens and environmental relatives. This suggests that most virulence genes, including T3S effectors, are equally important for fitness on crops and non-crop hosts and that pathogen populations in environmental reservoirs could be important sources of virulence genes for crop pathogens. Intriguingly, the frequency of a small number of virulence genes, and of some genes of yet unknown function, is significantly higher in crop pathogens compared to their frequency in environmental relatives. Therefore, these genes appear to play a particularly important role in crop disease emergence and/or for fitness in agricultural settings. These findings provide a new basis for an improved understanding of crop pathogen emergence and control.

## Supplementary Data

47 Supplementary File 1

## Supplementary Data

72 Supplementary File 2

## Supplementary Data

35 Supplementary File 3

## Supplementary Data

88 Supplementary File 4

## Supplementary Data

51 Supplementary File 5

## Supplementary Data

196 Supplementary File 6

## Supplementary Data

47 Supplementary File 7

## Supplementary Data

552 Supplementary File 8

## Supplementary Data

1504 Supplementary File 9

## Supplementary Data

55 Supplementary File 10

## Supplementary Data

24 Supplementary File 11
