# Supplementary figures and images for: Population-genomic insights into emergence, crop adaptation and dissemination of Pseudomonas syringae pathogens
Source: Microb Genom. 2016 Oct 21;2(10):e000089. doi: 10.1099/mgen.0.000089 (PMC5359406; doi:10.1099/mgen.0.000089)

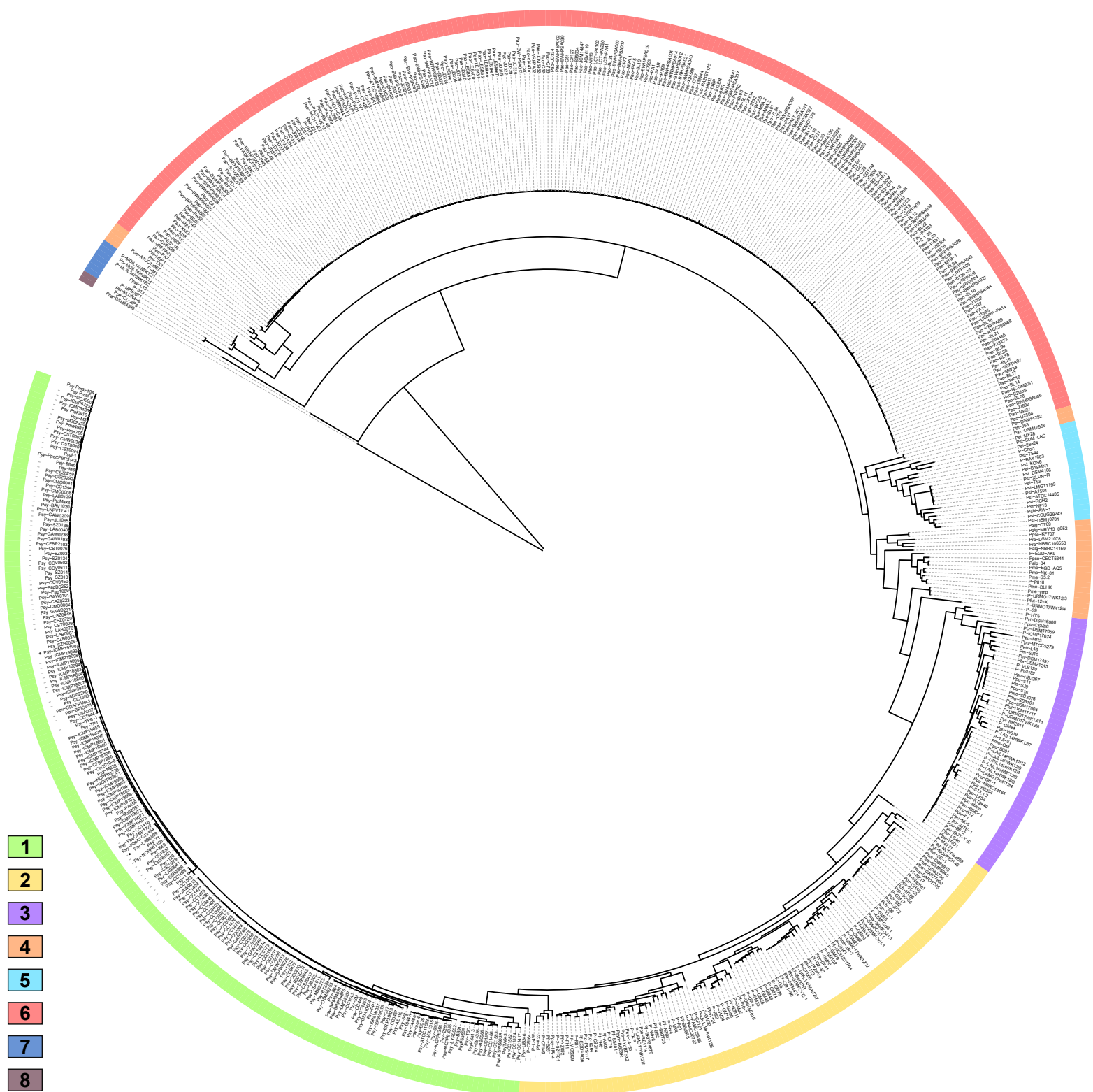

Supplement: Supplementary File 6 [file mgen-02-89-s006.pdf]

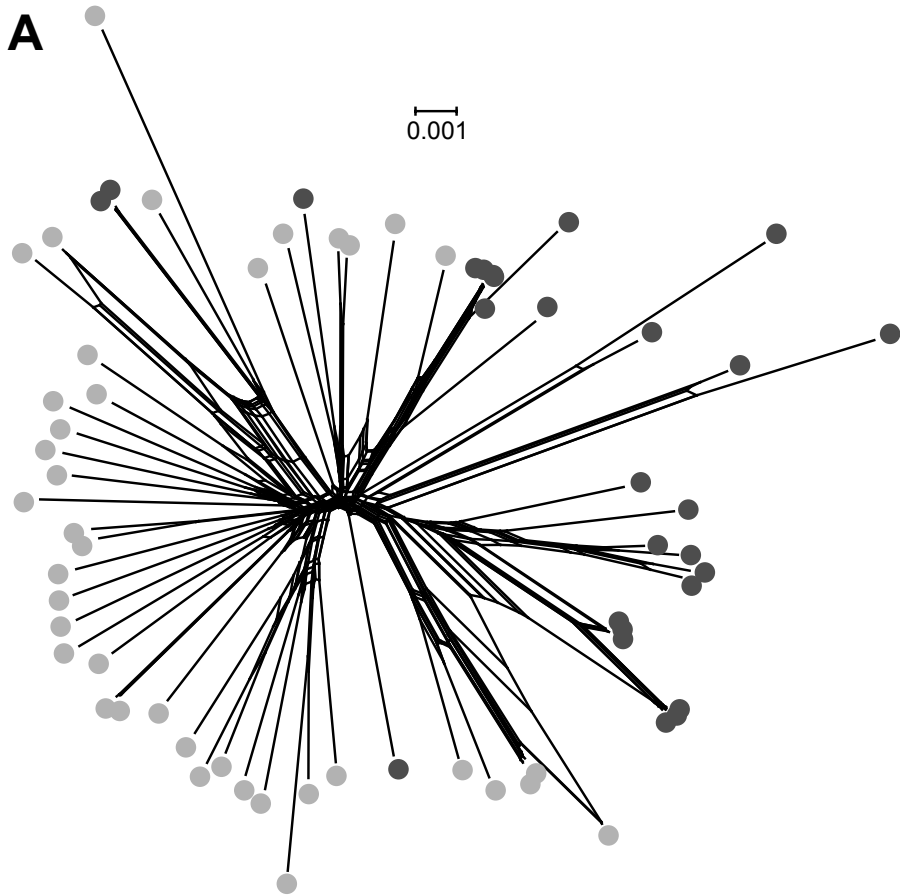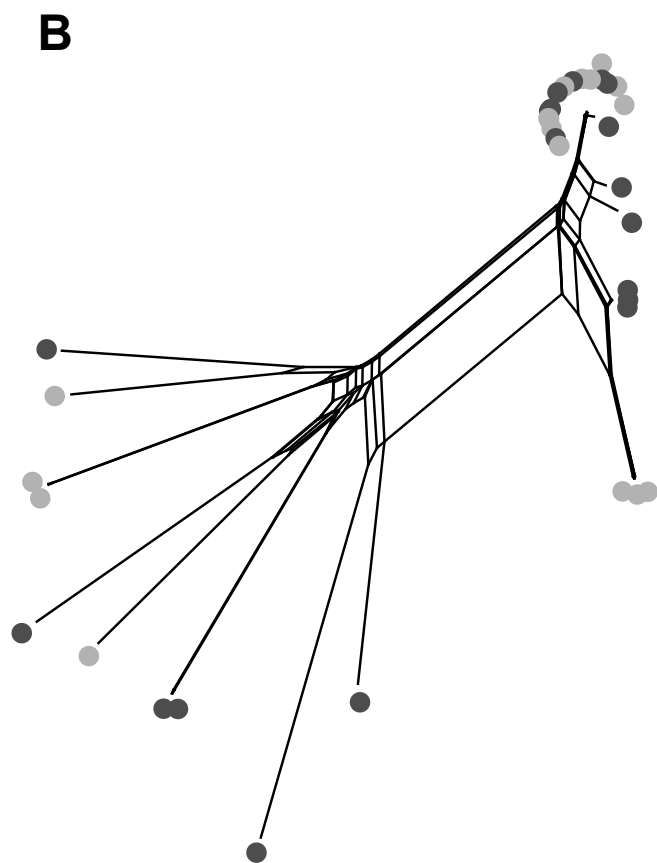

Supplement: Supplementary File 7 [file mgen-02-89-s007.pdf]

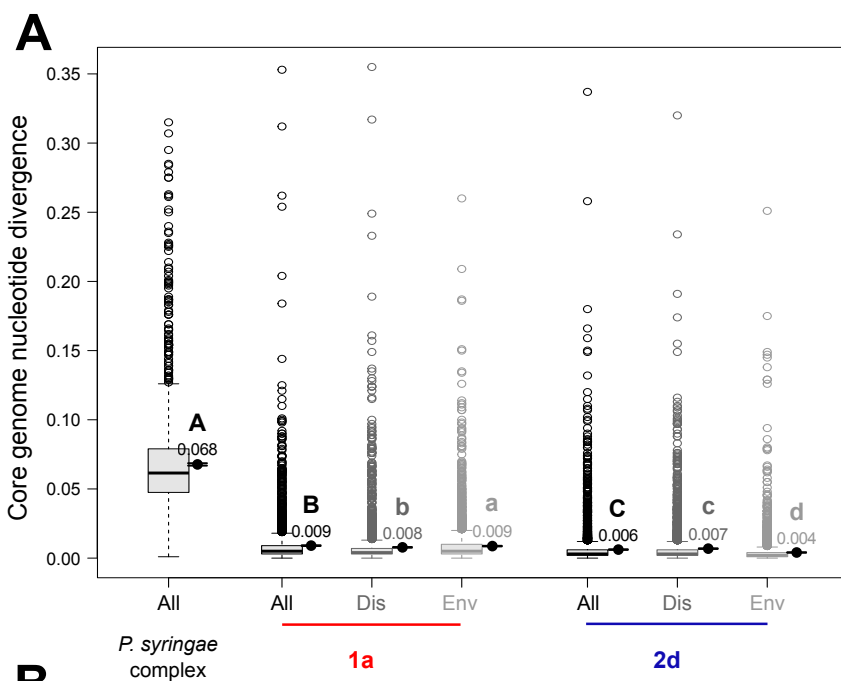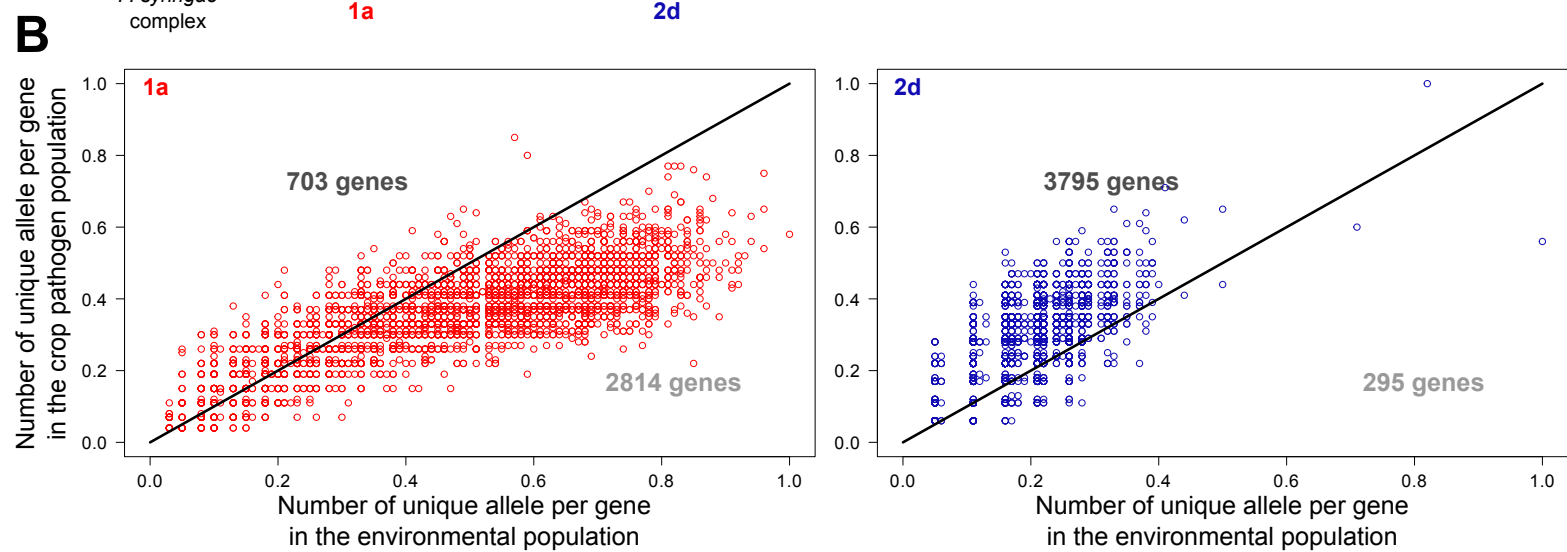

Supplement: Supplementary File 8 [file mgen-02-89-s008.pdf]

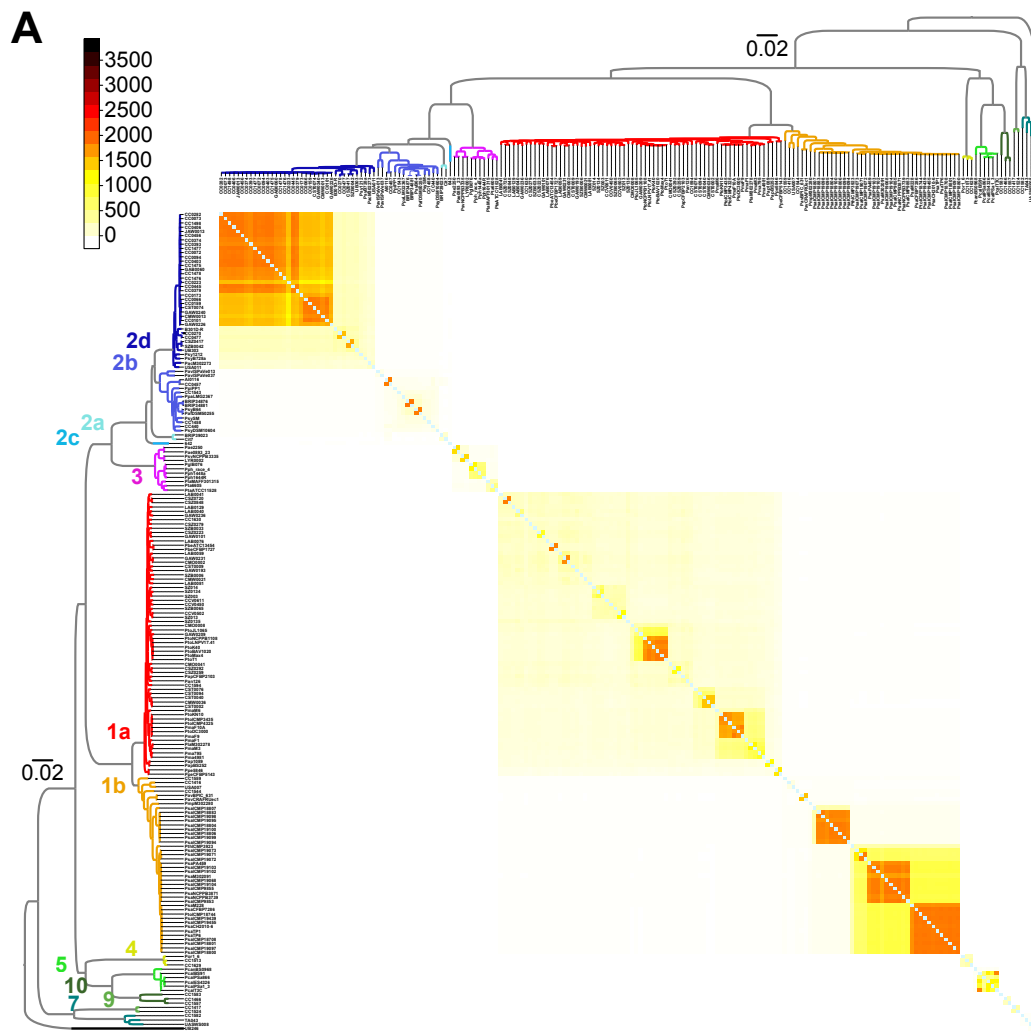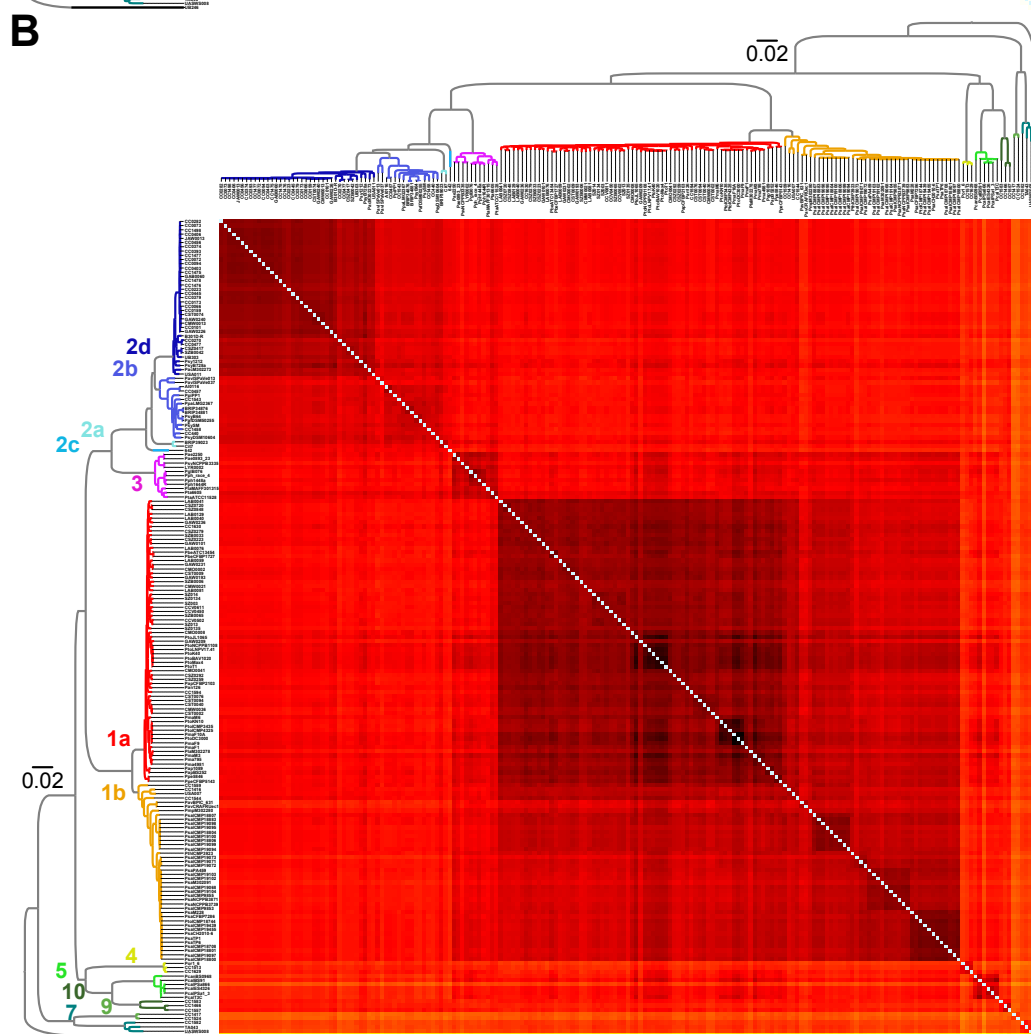

Supplement: Supplementary File 9 [file mgen-02-89-s009.pdf]

1a

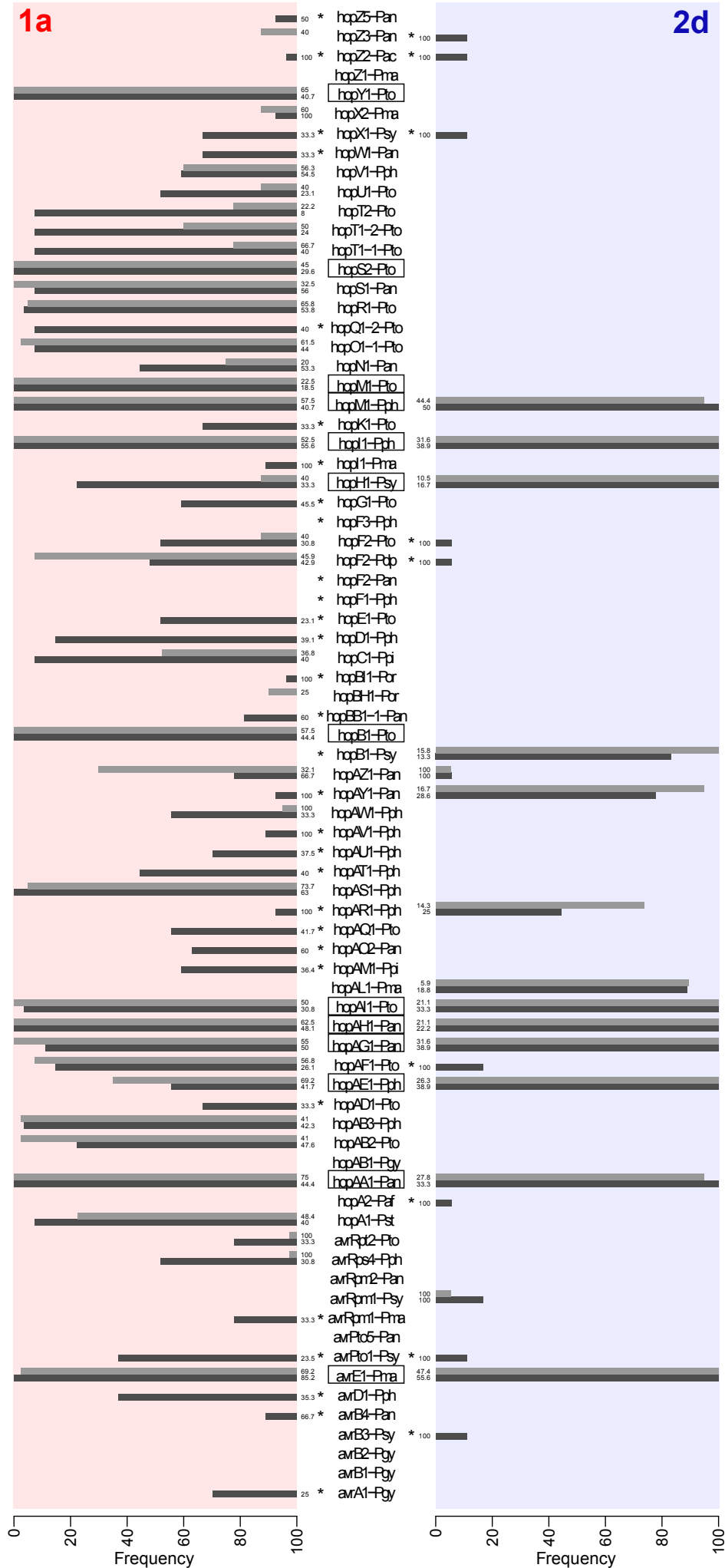

2d

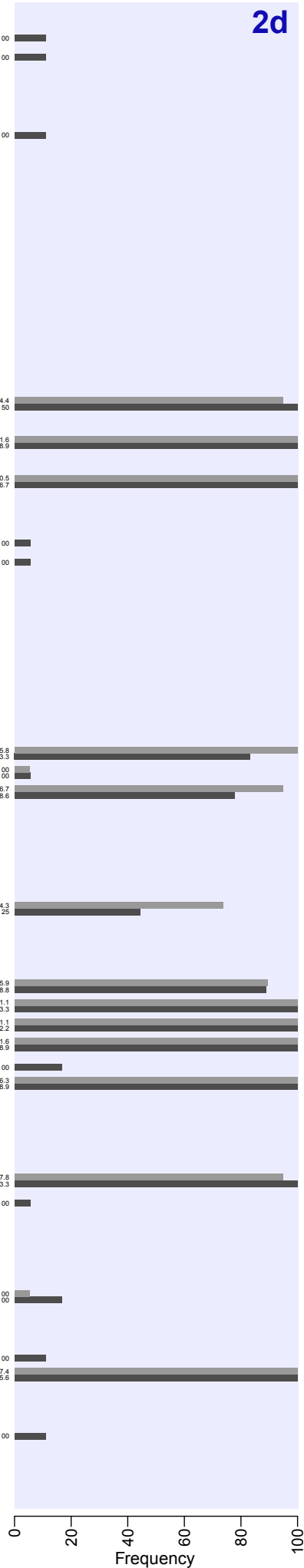

Supplement: Supplementary File 10 [file mgen-02-89-s010.pdf]

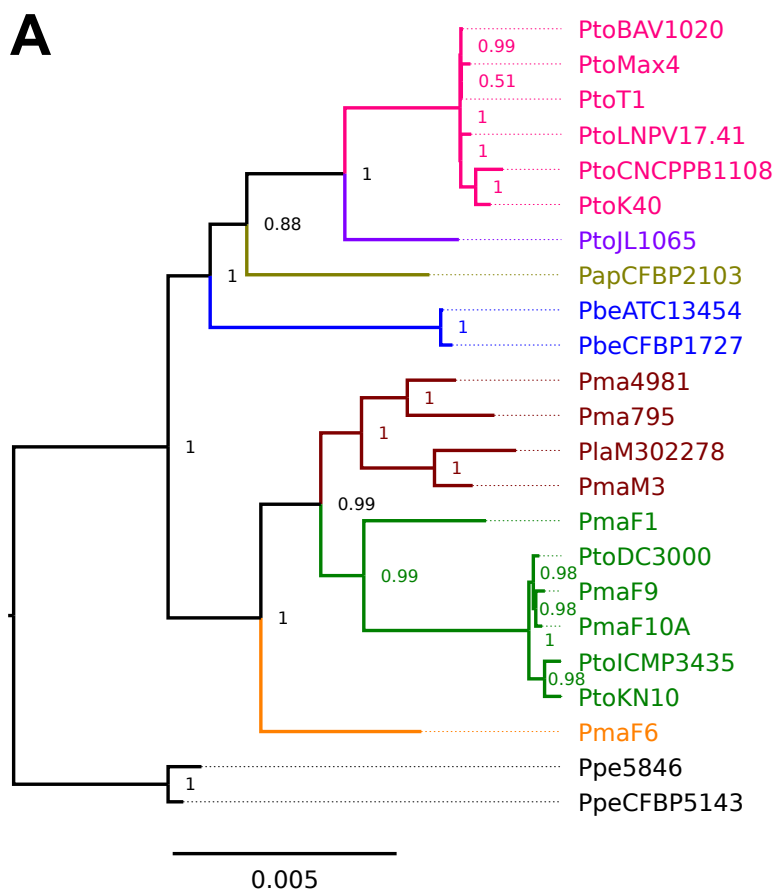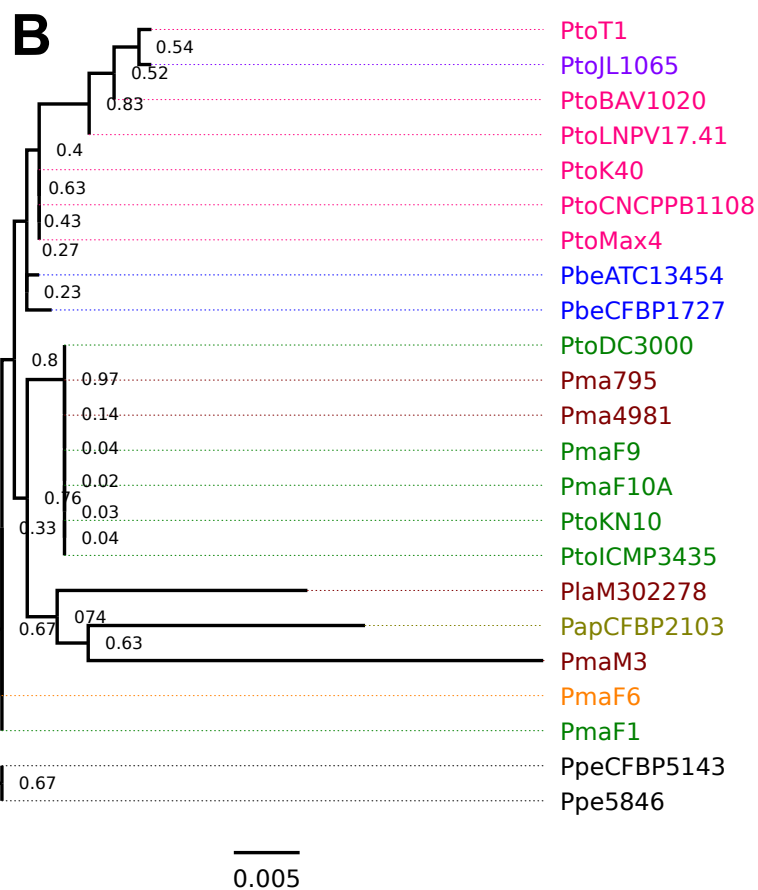

Supplement: Supplementary File 11 [file mgen-02-89-s011.pdf]
